# Single-application Radiofrequency Interruption in a Broad Isthmus Ventricular Tachycardia by Targeting the Longest Electrogram Visualized Using a New Customized Software (VEDUMap)

**DOI:** 10.19102/icrm.2021.120117S

**Published:** 2021-01-15

**Authors:** Filippo M. Cauti, Stefano Bianchi, Pietro Rossi

**Affiliations:** ^1^Arrhythmology Unit, Ospedale San Giovanni Calibita, Fatebenefratelli Hospital, Isola Tiberina, Rome, Italy

**Keywords:** Slow conduction, VEDUMap, ventricular tachycardia ablation

A 63-year-old woman with a diagnosis of arrhythmogenic right ventricular cardiomyopathy was referred to our department for recurrent monomorphic ventricular tachycardia (VT) with left bundle branch block and intermediate axis. A previous endocardial procedure was ineffective. After pericardial access, a detailed electroanatomical sinus rhythm map was obtained to verify areas of slow conduction and local abnormal ventricular activation **(Figure 1B)**. A confined spot of late potentials (LPs) was detected in an inferolateral aneurism. A marked deceleration zone in sinus rhythm was revealed at the base of the apex of the spot of LPs. VT was induced with a single extrastimulus and the full cycle length was recorded in the epicardium by the Advisor™ HD Grid Mapping Catheter, Sensor Enabled™ using the HD Wave acquisition algorithm **(Figure 1A)**. The VT isthmus revealed a broad path with inferior entrance and anterolateral exit in the epicardial base of the right ventricle. Thus, a novel ventricular map (VEDUMap) of electrogram (EGM) duration (unpublished data), which considered the duration of the EGM in a color-coded fashion (white = longest to purple = shortest) displayed with auto-color was created **(Figure 1C)**. The region of the prolonged EGM during VT corresponds to the slowest conduction in sinus rhythm. Radiofrequency (50 W at 43°C with an open irrigated catheter) (FlexAbility™ ablation catheter) was started at the white spot highlighted by the VEDUMap with sudden interruption of the tachycardia (green dot in **Figure 1) (Video 1)**.

After a single RF pulse, the VT was no longer inducible up to the fourth extrastimulus. A line of radiofrequency energy was delivered between the two VT isthmus boundaries. LPs were abolished and confirmed by remapping. The patient remained free from arrhythmia at 11 months of follow-up.

## Figures and Tables

**Figure 1:**
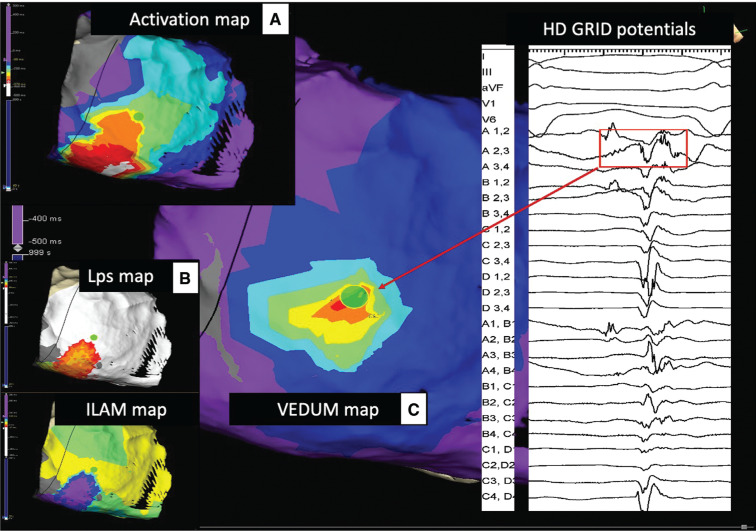
**A:** Activation map with first deflection detection shows a broad VT isthmus with an entrance on the inferior epicardial wall (white) and exit towards the right ventricular epicardial base. **B:** Sinus rhythm maps (local activation time map) with LPs map and isochronal late activation map. **C:** Ventricular map of EGM duration shows the crucial spot of VT interruption. The EGM recorded by the A2,3 bipoles (white color in the VEDUMap) in the EGM cover more than 60% of the diastolic phase. The green dot indicates the site of radiofrequency interruption.

**Video 1.** Single pulse VT interruption at the critical site.

